# Spousal Communication, Changes in Partner Attitude, and Contraceptive Use Among the Yorubas of Southwest Nigeria

**DOI:** 10.4103/0970-0218.51232

**Published:** 2009-04

**Authors:** Peter O Ogunjuyigbe, Ebenezer O Ojofeitimi, Ayotunde Liasu

**Affiliations:** Department of Demography and Social Statistics, Obafemi Awolowo University, Ile-Ife, Osun State, Nigeria; 1 Department of Institute of Public Health, College of Health Sciences, Obafemi Awolowo University, Ile-Ife, Osun State, Nigeria

**Keywords:** Counseling, decision-making, reproduction

## Abstract

**Objective:**

This paper highlights the relevance of spousal communication on males' attitude towards their partners' contraceptive use.

**Design:**

This was a cross-sectional study.

**Materials and Methods:**

Data for the study were obtained from a survey carried out in three states, Oyo, Osun, and Ondo, mainly inhabited by the Yorubas.

**Results:**

The results show that men have a significant role to play in the adoption of contraception. About 37% of the respondents reported joint decision making on when to have another child, 40.8% on whether to stop having children, and 44% on what to do to stop childbearing. Communication between a husband and wife on reproductive matters was also recognized as a factor that may influence male participation in family planning.

**Conclusion:**

This study has shown that the male partner may be highly motivated to obtain contraceptives. The results therefore suggest that male involvement in family planning should be encouraged through inter-spousal communication.

## Introduction

Though it is realized that the target group in family planning are the females and especially, women of childbearing age, it is averred that the male is an important factor with far reaching positive or negative implications for the practice.(1) In this context, the decision to have or not to have children is the male's and invariably his decision is usually in favor of having children, as more children further enhance his status as a man in society. Isiugo-Abanihe,(2) Raimi,(3) and Bruce(4) noted that male dominance is particularly profound in matters of reproduction. They generally view reproduction as their prerogative, an issue in which the compliance of their wives is taken for granted.

Men need information about contraceptive methods for women as well as about those for men. Well-informed men can use a method themselves or support their partners in using a method. They can also talk with their wives and cooperate in assessing their needs and choosing a family planning method.(5) Except for female prostitutes, men are likely to have more sexual partners than women. They have more control over condom use and are more likely to control the frequency of sexual relations and the possibility of abstinence within a relationship. Furthermore, the relatively high fertility level in Nigeria points to the need for a closer examination of the mechanisms of spousal communication about fertility decision-making among couples in different family settings. One factor deriving emphasis on the couple over the individual, as observed by Biddlecom and Fapohunda,(6) has been an increasing number of studies that demonstrate the influence of a man's preferences and power on reproductive outcomes such as contraceptive use,(7) childbearing,(2,8,9) and views about family planning.(10,11) Based on these studies and corroborating Becker's position,(12) one could argue that reproductive health programs that attempt to reach women will have a higher probability of success if they also involve the husband or at least encourage such involvement. Therefore, an understanding of the males' influence and the role they play in decision-making on contraceptive use can throw better light on mechanisms through which fertility reduction can be achieved.

## Materials and Methods

The data for this paper were derived from the survey conducted in three states of South Western Nigeria. These are Osun, Oyo, and Ondo, which are mainly inhabited by the Yorubas. The primary respondents are 600 married men aged 15-59 years old while their wives constitute the secondary respondents. In each state, the state capital and two adjacent rural areas were selected for the study. A multistage, stratified, random sampling design was used to select respondents from the towns. In the rural areas, selection of respondents was completed by a simple random sampling technique. However, the random selection was made in such a way that all the different parts of the locations were represented.

While a structured interview was employed to collect information on social, demographic, and reproductive-related variables that could be quantitatively measured, focus group discussions (FGDs) were used to collect additional information on the cultural practices of the people. For the purpose of analysis, the dependent variable was contraceptive use, which was related to a number of motivational, demographic, and socioeconomic independent variables.

Generally, the data collected were analyzed at three levels and each level required different analytic procedures. The first level involved an examination of the distribution of the respondents according to each of the selected characteristics. The second level involved the examination of the patterns of association between the dependent and independent variables. At the third level, multivariate analyses were employed to examine the interrelationships between the respondents' background characteristics and contraceptive use. Logistic regression models were employed to assess the association between some selected background variables like age, place of residence, religion, educational attainment, etc. and contraceptive use. Contraceptive use has a value of 1 if both partners reported use thus, indicating that both partners recognize they are adopting a method for the purpose of delaying or preventing pregnancy and zero (0) if only one or none of them reported use at the time of the survey. The results of the logistic regression models are presented as relative odds.

The reference category of each dichotomously measured independent variable has a value of one and the values for other categories are compared to that of the reference category. A value less than 1 implies that individuals in that category have a lower probability of reporting current use of contraceptives than individuals in the reference category. For continuously measured independent variable, a value less than 1 implies a less risk and a value greater than 1, greater risk of reporting current use of contraceptives as value of that variable increases.

## Results

### Sample characteristics

The socioeconomic characteristics of the respondents are presented in Table 1. The table shows that a majority of the respondents fell within the age range 25-39 years old (60.3%). Whereas males outnumbered females in the ages of 40 years and above, the females were more than males at the lower ages. A higher percentage of the respondents reside in urban areas (56.4%). More than 90% of the male population and 86.2% of the female population had received formal education; the highest being secondary for both male and female respondents. Marriage was largely universal and stable among the respondents. About 93% of the female respondents were still in their first marriage while about 74% of them were first wives of their husbands. Approximately 80% of both male and female respondents were engaged in some type of employment. However, the dominant occupation among the respondents, as shown in Table 1, was trading (38.1%) followed by farming and public/civil service with 20.9% and 16.4%, respectively. About 80% of the respondents professed to be Christian comprising 15.2% Catholic, 19.2% Protestant, and 45.5% belonging to another sect. About 18% were Muslims while the remaining belong to other religious groups.

**Table 1:** Background characteristics of respondents (percentage distribution)

Characteristics	Sex composition		
	
	Male (N=521)	Female (N=647)	Both genders (N=1168)
Age			
15–29 years old	7.1	39.3	23.5
30–44 years old	55.9	57.9	56.9
45+	36.9	2.8	19.9
Residency			
Urban	60.0	52.7	56.4
Rural	40.0	47.3	43.6
Education			
None	6.7	18.8	12.8
Primary	11.1	16.7	14.1
Secondary	57.8	46.0	51.9
Tertiary	20.0	16.7	18.2
Other	4.4	1.9	3.0
Marital status			
Married	92.9	92.7	92.8
Divorced	2.5	3.6	3.1
Widowed	4.5	3.6	4.0
Position among husband's wives			
1^st^ wife	-	73.4	-
2^nd^ wife	-	17.9	-
3^rd^ wife or higher order wife	-	8.9	-
Presently working?			
Yes	72.5	87.2	79.8
No	28.5	12.8	20.1
Occupation			
Farming	18.6	23.3	20.9
Trading	34.5	41.7	38.1
Public/civil servant	21.3	11.6	16.4
Professional	9.5	5.3	7.4
Artisan	11.8	8.6	10.3
Other	4.3	9.5	6.9
Religion			
Catholic	17.8	13.0	15.2
Protestant	20.0	18.5	19.2
Other Christian	40.0	50.0	45.5
Islam	20.0	16.7	18.2
Other	2.2	1.9	2.0
Total	100.0	100.0	100.0

### Men's influence in reproductive issues

Table 2 shows that in almost all cases of reproductive issues, husbands and wives reported joint spousal decision-making. The marginal frequencies, however, show that men are less likely than their wives to report joint decision-making and are more likely to report that they alone usually take decisions. Thus corroborating Isiugo-Abanihe's findings in an earlier study that indicated that 40% of men and more than 50% of women said decisions about their family size was a jointly taken decision.(1) This response must, however, be seen within the context of the Yoruba traditional society where the man is expected to have absolute control of his household and the woman is expected to respect whatever decision the husband takes. The desire to boost his ego and show that he is in control could make a man report that he alone takes decision (even when the issues are discussed with the wife); also the need to portray that the woman is well cultured through deference to her husband may make her report that only the husband takes decisions on these issues. This situation is more likely among respondents with lower levels of education and those in the rural areas. Despite these high levels of discordance in partners' responses, a significantly high proportion of couples still reported joint-decision making. About 37% of the respondents reported joint-decision making on when to have another child, 40.8% of the respondents reported joint-decision making on whether to stop having children, and 44% of the respondents reported joint-decision making on what to do to stop childbearing. The sum of the principal diagonal elements, which indicate agreement between partners' responses, indicate that 52.3%, 53.4%, and 55% of partners gave similar responses on who makes decisions on when to have another child, whether to stop childbearing, and what to do to stop childbearing.

**Table 2:** Husband and wife's responses on who takes decisions on reproductive issues↑

Husband	Wife			
	
	Husband only	Wife only	Husband and wife	Other
When to have another child				
Husband only	**15.1**	2.7	19.5	2.1
Wife only	0.6	**0.9**	1.4	0.2
Husband and wife	12.8	3.5	**37.2**	-
Other	1.1	0.2	2.2	**0.5**
Whether to stop childbearing				
Husband only	**10.7**	3.4	16.4	1.8
Wife only	1.1	**0.9**	0.6	0.1
Husband and wife	11.8	2.5	**40.8**	0.6
Other	4.0	0.6	3.7	**1.0**
What to do to stop childbearing				
Husband only	**7.4**	7.7	15.8	1.5
Wife only	0.5	**2.1**	3.6	0.2
Husband and wife	4.6	4.4	**44.0**	0.3
Other	1.7	0.9	0.9	**1.5**

↑ This table shows the marginal frequencies which were used to explain the pattern of decision-making among couples. The marginal frequencies show the strength of joint decision-making among the couples. For instance, 15.1 percent of the couples agreed that it is only the husband that can take decision on when to have another child as against 0.9 percent among those who are of the opinion that such decision can be taken by the wife only.

### Attitude to family planning

Many men appear ready to change their reproductive behavior and are willing to participate more in reproductive health activities. However, some, for certain reasons (e.g., health concerns, side effects, or want of children) may oppose such participation. Respondents were asked if they approve or disapprove of the statement that “many couples do something to delay or prevent a pregnancy so that they can have just the number of children that they want and have them when they want them.” Approximately 63% of the men compared with just 35.7% of women would give consent to the use of family planning [Table 3]. This is in spite of the claim by most of the respondents that they discuss family planning issues. At least 50% of the women and 38.1% of the men indicated that they had discussed family planning matters with their spouses on three or more occasions. About 36% of the respondents gave an indication that their spouse would not stop them from using family planning methods; however, we discovered that more of the male respondents as compared with the female respondents (37.3% males vs. 35.5% females) could ascribe to this claim. Table 3 shows that more than 30% of the respondents had discussed the family planning issue with other persons aside from the spouses.

**Table 3:** Percentage distribution of respondents by gender and attitude to family planning and their counseling status

Attitude	Male	Female	Total
Approval of family planning			
Approved	62.7	35.7	50.5
Disapproved	7.8	26.2	16.1
Don't know	29.5	38.1	33.3
How often do you talk about family planning?			
Once	17.3	33.3	24.5
Twice	32.7	28.6	30.9
Three/More	38.1	50.0	44.7
Does your partner agree with you using family planning?			
Yes	37.3	35.5	36.3
No	62.7	64.5	62.6
Ever discussed with any other person aside from spouse			
Yes	31.3	34.2	32.6
No	68.7	65.8	67.4
Ever counseled on family planning?			
Yes	38.6	71.2	53.7
No	61.4	28.8	47.3
Counseling status of partners			
Both husband and wife counseled	-	-	31.5
Only one partner counseled	-	-	37.2
No partner counseled	-	-	21.3
Total	100.0	100.0	100.0

### Family planning information and counseling

Information on counseling was obtained by asking each respondent to state if he/she had ever been counseled on family planning. The question was informed, among others by our knowledge of the activities of community-based distributors of family planning methods and other groups with respect to the provision of family planning information, education, and counseling services in the study area. Table 3 shows that with respect to family planning information, education, and counseling, the husbands have a lower exposure rate. The significant impact of counseling on contraceptive use is worth noting, especially the implications for the participation of men in family planning. Men are more likely to take part in family planning once the needs for family planning are made clear to them.

### Current use of contraceptives

The results of the logistic regression models are presented as relative odds in Table 4. Education, age, when to stop childbearing, and the number of surviving children were found to have a significant impact on contraceptive use. The impact of education is particularly pronounced when none of the partners had below a secondary school education. Men with female partners below 25 years of age are also significantly more likely to use or report use of a modern contraceptive. The significant net impact of communication on contraceptive use is worth noting, especially the implications for the participation of men in family planning. The result draws attention to the possibility that men can actually use or support their partners' use of a contraceptive if they are given adequate information, education, and communication (IEC) on the need and ways to regulate fertility. This confirms the findings of the previous studies by Ogunjuyigbe(13) and Feyisetan and Bamiwuye,(14) which recognized the importance of counseling in contraceptive use. Whether partners take joint decisions on when to stop childbearing and the number of surviving children have significant positive association with the probability that a man would report current use of a modern method after controlling for other factors.

**Table 4:** Logistic regression result of the effect of the current use of modern methods

Background characteristics	Odds ratio
Residence	
Urban	1.472
Rural (RC)	1.000
Age	
1 5-24 years old	4.235*
25-34 years old	2.562
35 years old and above (RC)	1.000
Joint education of partners	
Both had primary or below	1.000
One had primary or below, the other secondary or above	1.265
Both had secondary	3.477*
At least one had post-secondary, the other secondary	4.512**
Desired family size	
Both partners want more (RC)	1.000
Husband more, wife no more	0.492
Husband no more, wife more	1.564
Both want more	0.519
Religion	
Protestant	0.673
Catholic	0.097*
Other Christian	0.469
Islam	1.000
Joint decision on contraception	
Yes	1.115
No	1.000
Joint decision on number of children	
Yes	3.217
No	1.000
Joint decision on when to stop childbearing	
Yes	0.678
No	1.000
-2 log likelihood	352.172
Model Chi-square	126.616

** Significant at *P*≤0.01

* Significant at *P*≤0.05; RC = reference category

## Discussion and Conclusion

The effects of a number of factors on the current use of contraceptives were highlighted in this paper. Communication variables such as decisions about family size and family planning as well as spouse's perception of partner's approval of family planning all have significant impact on the current use of contraceptives. This study revealed that marital partners who discuss and take joint decisions on what to do to delay or stop childbearing are more likely to use contraceptives than their counterparts who have not discussed the issue. However, the significance of the impact decreases while controlling other factors. The finding that joint-decision making was an important explanatory variable in current contraceptive use shows that men have a role in the adoption of contraception. We equally noticed that there is a highly significant impact of family planning counseling on contraceptive use, especially its implication for participation of men in family planning. Findings in the study tend to show that men are more likely to take part in family planning once the needs for family planning are made clear to them.

Finally, the relatively high fertility levels in Nigeria call for a closer examination of the mechanisms of fertility decision-making among couples in different family settings. But since the husband is very important in family decision-making, it is essential that the male should be adequately informed on population issues. This is necessary in order to increase his understanding and enhance his encouragement and support for his wife who is the main target of contraceptive innovation. Male acceptance of contraception is at least as effective in preventing pregnancy as is female acceptance, and perhaps more so as reflected in the higher continuation and use-effectiveness rates.(15) The male partner may be highly motivated to obtain contraceptives. This may be related to his desire to control the use and choice of the contraceptive or to assure himself that the objective of avoiding an unwanted pregnancy is achieved, particularly in an extramarital relationship.
